# Mycobacterial Virulence Factors: Surface-Exposed Lipids and Secreted Proteins

**DOI:** 10.3390/ijms21113985

**Published:** 2020-06-02

**Authors:** Angel Ly, Jun Liu

**Affiliations:** Department of Molecular Genetics, University of Toronto, Toronto, ON M5G1M1, Canada; angel.ly@mail.utoronto.ca

**Keywords:** *Mycobacterium tuberculosis*, mycobacteria, virulence factors, pathogenesis, type VII secretion systems

## Abstract

The clinically important *Mycobacterium tuberculosis* (*M. tb*) and related mycobacterial pathogens use various virulence mechanisms to survive and cause disease in their hosts. Several well-established virulence factors include the surface-exposed lipids in the mycobacterial outer membrane, as well as the Esx family proteins and the Pro-Glu (PE)/ Pro-Pro-Glu (PPE) family proteins secreted by type VII secretion systems (T7SS). Five ESX T7SS exist in *M. tb* and three—EsxA secretion system-1 (ESX-1), ESX-3, and ESX-5—have been implicated in virulence, yet only the structures of ESX-3 and ESX-5 have been solved to date. Here, we summarize the current research on three outer membrane lipids—phthiocerol dimycocerosates, phenolic glycolipids, and sulfolipids—as well as the secretion machinery and substrates of three mycobacterial T7SS—ESX-1, ESX-3, and ESX-5. We propose a structural model of the *M. tb* ESX-1 system based on the latest structural findings of the ESX-3 and ESX-5 secretion apparatuses to gain insight into the transport mechanism of ESX-associated virulence factors.

## 1. Introduction

The host–pathogen relationship between humans and *Mycobacterium tuberculosis* (*M. tb*) has been evolving for approximately 50,000 to 70,000 years [1], and its persistence as an infectious pathogen has led to 10 million new cases of tuberculosis (TB) and 1.2 million TB-related deaths in 2018 [2]. The clinically important pathogen *M. tb* belongs to the *Mycobacterium* genus, which is characterized by bacteria that exhibit slow-growth, high GC genome, and a distinguishingly thick cell wall [3]. TB in humans and animals results from infection by the *M. tb* complex, which consists of *M. tb, M. africanum, M. bovis*, *M. canetti*, *M. caprae*, *M. microti*, *M. mungi*, *M. orygis*, *M. pinnipedii* and *M. suricattae* [4,5,6,7]. These pathogens infect and multiply in alveolar macrophages to cause pulmonary TB disease [8], but they can also lead to extrapulmonary disease when bacteria disseminate outside of the lungs, such as to the central nervous system and/or lymph nodes [9]. Another class of clinically relevant mycobacteria includes non-tuberculosis mycobacteria such as the *M. avium* complex [10,11], *M. marinum* [12,13], and *M. ulcerans* [14], which are opportunistic pathogens that can cause disease in immunocompromised individuals. The complexities of mycobacterial physiology, morphology, and virulence mechanisms render pathogens in the *Mycobacterium* genus a difficult target of study for the development of effective antimycobacterial therapies.

**Figure 1:**
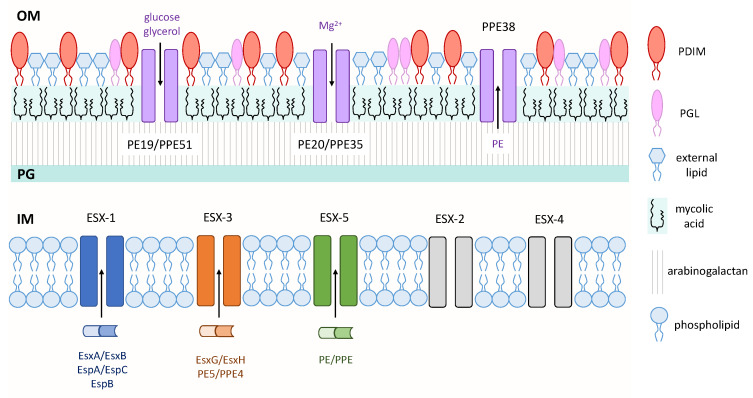
Virulence factors of mycobacteria. Three type VII secretion systems—ESAT-6 secretion system-1 (ESX-1), ESX-3, and ESX-5—secrete proteins across the IM. Pro-Glu (PE)/Pro-Pro-Glu (PPE) small-molecule selective channels transport nutrients and proteins across the OM. Various mycobacterial cell wall lipids are depicted on the outer leaflet of the OM. Inner membrane (IM); peptidoglycan (PG); outer membrane (OM).

The success of mycobacterial pathogens in causing disease involves various mechanisms that enable colonization, replication, and survival in their host, thus mycobacterial virulence factors are typically defined as bacterial genes or cellular components that enable their overall survival in the host. If a deletion or loss of any particular gene or cellular component impairs bacterial growth in the host, it is considered a virulence factor. Some of these factors have been identified by genomic, biochemical, and functional analysis of *M. tb* and related mycobacterial pathogens, therefore several of these genes or cellular components that have well-recognized roles in virulence will be the focus of this review (Figure 1).

## 2. Localization in the Outer Membrane: Surface-Exposed Lipids and PE/PPE Family Proteins

The mycobacterial cell wall is unique in that it contains a layer of arabinogalactan that is covalently linked to a large amount of long-chain (C_60_–C_90_) fatty acids called mycolic acids. This layer of mycolic acids forms the inner leaflet of the mycobacterial outer membrane, while various complex lipids, including glycolipids, form the outer leaflet, which together form a thick hydrophobic barrier that is often difficult to penetrate with standard approaches [15] (Figure 1). This characteristic of mycobacteria is a contributing factor to the ineffectiveness of many antibiotics against this genus. While this section will discuss three outer membrane lipids that have well-established roles as virulence factors, the Pro-Glu (PE)/Pro-Pro-Glu (PPE) family proteins will also be discussed since these proteins exhibit outer membrane localization, have roles in virulence, and may provide a newly-identified means for nutrient and/or protein transport across this hydrophobic barrier.

### 2.1. Phthiocerol Dimycocerosates (PDIMs) and Phenolic Glycolipids (PGLs)

Phthiocerol dimycocerosates (PDIMs) and phenolic glycolipids (PGLs) are structurally related complex lipids in the mycobacterial outer membrane that have been shown to be critical for mycobacterial virulence (Figure 1). PDIMs are methyl-branched fatty acid-containing lipids that are present only in pathogenic mycobacteria such as *M. tb, M. bovis* and *M. marinum* [16]. PDIMs were first implicated in virulence using signature-tagged transposon mutagenesis which identified *M. tb* PDIM mutants that were attenuated in mice [17,18]. Subsequently, PDIMs have been shown to mediate receptor-dependent phagocytosis of *M. tb* [19], contribute to the cell wall permeability barrier [20], and protect against reactive nitrogen intermediates in activated macrophages [21]. More recently, it was shown that PDIMs also contribute to host cell escape and necrosis [22]. As many virulence factors do not function independently, PDIM-mediated virulence has been recently demonstrated to work in concert with EsxA secretion system-1 (ESX-1), a type VII secretion system in mycobacteria. An EsxA-deficient mutant in a PDIM-deficient background of *M. tb* showed a decreased ability of EsxA to induce phagosomal rupture in human monocyte-derived macrophages, which may have resulted from changes in membrane rigidity [23].

PGLs are also restricted to pathogenic mycobacteria except that in *M. tb*, only a subset of clinical isolates produces this glycolipid [16]. The phenolic glycolipid 1 (PGL-1) of *M. leprae*, the causative agent of leprosy, has been implicated in the invasion of host phagocytic cells [24]. Using a recombinant *M. bovis* BCG strain that produces PGL-1, it was shown that the infectivity of this recombinant strain was increased, and it also had a growth advantage in human monocyte-derived macrophages compared to the parent BCG strain. The production of PGL-1 also increased the capacity of recombinant BCG to use the complement receptor 3 pathway for host macrophage invasion. Another study implicated the PGLs of *M. marinum*, a close relative of *M. tb* that causes disease in fish and amphibians, in bacterial virulence through its involvement with the host chemokine receptor 2 pathway that promoted macrophage recruitment [25]. These host–pathogen interactions highlight the role of PGLs as a key player in mediating host-specific responses at the host–bacterium interface.

PGLs have also been implicated in dampening the host immune response by inhibiting the release of pro-inflammatory cytokines, which has been associated with a hypervirulent phenotype of certain *M. tb* clinical isolates such as the W-Beijing family [26,27]. The W-Beijing family of *M. tb* strains has gained scientific interest in its ability to cause outbreaks, which includes the infamous multidrug-resistant outbreak among HIV-infected individuals during the 1990s [28]. BALB/c mice that were infected with W-Beijing *M. tb* exhibited hypervirulence compared to 12 other *M. tb* strains and *M. tb* H37Rv [29]. One hypothesis to explain this unique endemic property of W-Beijing concerns the differences between *M. tb* strains in their ability to synthesize PGL-tb. The Beijing lineage is the only family of *M. tb* strains that possesses an intact *psk1-15* sequence required for PGL-tb synthesis [30]. However, the ability to synthesize PGL-tb may only be important in the W-Beijing *M. tb* strain, as ectopic expression of PGL-tb in *M. tb* H37Rv was unable to alter its virulence in infected mice and rabbits [27]. It was also found that this strain of *M. tb* synthesizes high levels of triglycerides that coincides with an upregulation in the expression of the DosR dormancy regulon, which has been thought to confer an adaptive advantage for survival in dynamic environments encountered within the host [31]. Together, these studies suggest that PGLs are involved in the enhanced virulence of W-Beijing *M. tb*, though it is likely that PGLs may act in concert with other virulence factors to account for its high incidence of outbreaks.

The critical role of PDIMs and PGLs in virulence has also been demonstrated in *M. marinum* [32] and *M. bovis* [33]. It was shown that PDIM sand PGLs in *M. marinum* work in a concerted fashion to recruit permissive macrophages while restricting macrophages with high bactericidal activities, which favor mycobacterial survival and replication in the host [24]. Given that PDIMs and PGLs play important roles in host–pathogen interactions, it is of significance that three BCG strains—BCG-Japan, -Moreau, and -Glaxo—are naturally deficient in the production of PDIMs and PGLs [34]. The loss of PDIMs and PGLs production in these three BCG strains originated from independent mutations that arose during the in vitro evolution of BCG strains [35,36]. Interestingly, the loss of PDIMs and PGLs in BCG-Japan, -Moreau and -Glaxo correlates with their superior safety records in clinical studies over other BCG strains [34,37,38]. Consistently, BCG-Japan, -Moreau and -Glaxo are among the most attenuated strains of BCG in SCID mice models of infection [39], while a PDIM- and PGL-deficient strain of BCG-Pasteur exhibited reduced virulence in SCID mice [40]. These findings have important implications for future vaccine development, particularly for the construction of recombinant BCG.

### 2.2. Sulfolipids (SLs)

Surface-exposed sulfolipids (SLs) represent a family of sulfated acyl trehaloses in mycobacteria that have been attributed virulent properties by numerous in vitro and in vivo studies. SLs are categorized into two classes—SL-I and SL-II—based on their abundance from thin-layer chromatographic experiments [41]. Studies on the biosynthetic pathway of SLs have also identified essential enzymes and transporters required for these outer membrane lipids of *M. tb* [42,43]. Although it was later found using electrospray ionization mass spectrometry that SL-II, rather than SL-I, represented the major SL species in *M. tb* [44], many studies have already identified and characterized the virulent properties of SL-I.

SLs were first identified as a virulence factor by its ability to inhibit phagosome–lysosome fusion and acidification in infected murine macrophages [45]. In a more recent study that screened transposon mutants of a virulent clinical isolate of W-Beijing *M. tb* to identify molecular mechanisms of host immune evasion, it was found that a tetraacylated SL was involved in suppressing toll-like receptor 2 (TLR2) signaling [46]. As previous work identified that the *mmpL8* gene encodes a SL transporter [43], the *M. tb mmpL8*::Tn mutant exhibited increased NF-κB activation and induced stronger production of IL-8 in human THP-1 cells. These effects were mediated through increased TLR2 signaling, as it was subsequently found that SLs can act as competitive antagonists of TLR2 to inhibit the formation of TLR2/TLR1 or TLR2/TLR6 heterodimers to suppress the activation of macrophages, leading to a dampened innate immune response.

Aside from interactions with specific surface receptors on human macrophages, it remains unknown whether SLs can influence the dynamics of host cell membranes since SLs are located at the host–bacterium interface. It was recently found that SL-I mediates the remodeling of THP-1 cell membranes in a time and concentration-dependent manner that resulted in increased host membrane fluidity and altered membrane-associated signaling [47]. This evidence supports the finding that SLs inhibit the formation of TLR2/TLR1 and TLR2/TLR6 heterodimers in the host cell membrane [46]. These findings collectively form a putative framework of the mechanism of host cell membrane modulation by mycobacterial surface-exposed lipids. 

### 2.3. PE/PPE Family Proteins

The *Mycobacterium* genus-specific PE/PPE proteins share conserved N-terminal motifs that include Pro-Glu (PE) or Pro-Pro-Glu (PPE) residues and a highly variable C-terminus, many of which are secreted by type VII secretion systems and are present mostly in pathogenic mycobacteria [48]. Examining the *M. tb* H37Rv genome led to the identification of 99 *pe* and 69 *ppe* genes, which comprise nearly 10% of its coding potential [48]. In addition to their isolated location in the *M. tb* genome, a pair of *pe/ppe* genes also flank the *esxA-esxB* family genes in each *esx* locus, with the exception of *esx-4* [49]. In nonpathogenic *M. smegmatis*, two pairs of *pe/ppe* genes have been identified corresponding to the orthologues of *Rv3872/3* from the *esx-1* locus and *Rv0285/6* from the *esx-3* locus in *M. tb* [49], which may represent the most ancestral genes of the PE/PPE protein family. This also suggests that they were likely associated with the ESX-1 system first, then co-duplicated and further expanded independently of the ESX secretion systems. The only *M. tb* PE/PPE protein structures solved to date have been the PE25/PPE41 dimer [50] and its complex with ESX secretion-associated protein G5 (EspG5) [51], as well as the PE8/PPE15 dimer bound to EspG5 [52]. Despite their abundance in *M. tb*, the mechanisms of many PE/PPE proteins remain elusive due to their high sequence homology and unstable, insoluble recombinant protein structures.

These secreted PE/PPE proteins are associated with the mycobacterial outer membrane and have been thought to play a role in interacting with the host immune system [53]. Upon closer inspection of the virulence potential of some PE/PPE proteins, PE11 is expressed in pathogenic mycobacteria but absent in *M. smegmatis* [54]. PE11 is upregulated in *M. tb* during stress conditions and macrophage infection, and expression of PE11 in recombinant *M. smegmatis* revealed its role in shaping cellular morphology through an increase in cell envelope hydrophobicity. PE11-expressing *M. smegmatis* exhibited higher bacterial burden in the lung, liver, and spleen in infected mice, which suggests PE11 as a possible virulence factor in *M. tb* through its modulatory effects on the composition of the cell wall. 

In the closely related pathogen *M. marinum*, the *mag24* and *mag85* genes were identified as homologues of the *M. tb* PE_PGRS subfamily of PE proteins, and disruption of *mag24* led to reduced bacterial load in J774 macrophages [55]. *M. marinum* also encodes WhiB4, an iron-sulfur containing transcription factor, that has been implicated in the regulation of PE_PGRS gene expression [56]. The *whiB4* deletion mutant had reduced bacterial load in RAW macrophages and exhibited attenuation in zebrafish, which collectively demonstrate PE/PPE proteins as a virulence factor across pathogenic mycobacterial species.

Importantly, a recent publication identified a novel role for the PE/PPE proteins as outer membrane small-molecule channels that enable nutrient uptake across this impermeable barrier [57]. From a library of putative small-molecule antibiotics against *M. tb*, 3,3-bis-di(methylsulfonyl)propionamide (3bMP1) significantly reduced the growth of *M. tb* and colonies that showed resistance to 3bMP1 were found to harbor mutations in the *ppe51* gene. These PPE51 mutants exhibited impaired growth on propionamide, glucose and glycerol, and were also later found to harbor a mutation that inhibited the synthesis of PDIMs. It was shown that PPE51 acts with PE19 to form a selective channel on the outer membrane that enables the uptake of carbon sources into the periplasmic space. This porin-like characteristic is not exclusive to PE19/PPE51, as PE20/PPE31 was shown to mediate the uptake of magnesium. Another recent finding related to PE/PPE-mediated transport was that mutations in the *ppe38* gene completely blocked the secretion of more than 80 PE_MPTR and PE_PGRS family proteins, which represent two large subsets of ESX-5 substrates [58]. It is tempting to propose that PPE38 may form, or be a component of, an outer membrane channel similar to the abovementioned PE/PPE channels to enable the secretion of PE proteins (Figure 1). These recent and novel findings have revolutionized the field of *M. tb* research, as a canonical porin has never been found in the outer membrane of *M. tb* despite the obvious need for nutrient transport in hostile environments within the host.

## 3. Localization in the Inner Membrane: ESX Type VII Secretion Systems (T7SS)

Protein secretion plays an important role in the ability of bacteria to adapt to changing environments. Many bacteria require the translocation of enzymes, proteins and toxins across its membrane, several of which are involved in pathogenesis. Mycobacteria use an essential general secretion pathway called the Sec secretion system to translocate unfolded proteins with an N-terminal signal sequence across the inner membrane [59]. Bacteria in this genus also use a sec-independent system called the twin-arginine transporter pathway to translocate folded proteins across the inner membrane [59]. In addition to these secretion systems, *M. tb* and related pathogenic mycobacteria encode five type VII secretion systems (T7SS), designated EsxA secretion system-1 (ESX-1) to ESX-5. Three of them—ESX-1, ESX-3 and ESX-5—are required for the full virulence of *M. tb*, while the roles of ESX-2 and ESX-4 remain unknown. A major function of these ESX systems involve protein transport across the inner membrane, which has been demonstrated for ESX-1, ESX-3 and ESX-5, but not for ESX-2 and ESX-4. While these T7SS have been extensively reviewed elsewhere [60], we will focus on some of the recent progress on the structural organization and transport mechanism of these unique systems.

### 3.1. ESX-5

The ESX-5 secretion system is the most recently evolved T7SS and is only found in slow-growing mycobacterial species [61]. ESX-5 was the first T7SS that had its structure solved [62], which provided valuable insight into the mechanism of secretion by these unique systems (discussed below). Much knowledge of the function of ESX-5 was gained from studies of *M. marinum*. 

Analysis of *M. marinum* ESX-5 transposon mutants revealed that ESX-5 is required for the secretion of various PE/PPE proteins, such as the heterologously expressed PPE41 of *M. tb* or PE and PPE proteins belonging to the PE_PGRS and PPE_MPTR subgroups [63,64]. It was revealed that a functional ESX-5 system is required for cell wall integrity, secretion of EsxN and PPE41, and the full virulence of *M. tb* [65]. A key feature of these Esx substrates is that they belong to the WXG100 protein superfamily. Proteins in this group are approximately 100 amino acids in length and contain a conserved Trp-X-Gly (WXG) motif and a helix-turn-helix domain that is followed by an YxxxD/E secretion motif required for export [66]. Several other studies have attributed similar roles to ESX-5 in modifying cell wall permeability for nutrient uptake [67], as well as identifying the role in virulence of its PE and PPE substrates [68]. ESX-5 was shown to facilitate the cell-to-cell spread of *M. marinum* in infected macrophages, a function that is shared by ESX-1 [63]. However, ESX-5 does not complement the loss of virulence caused by ESX-1 deletion, suggesting that these two ESX systems play distinct roles in virulence [60]. 

While characterizing ESX-5a, a duplicated *esx-5* gene cluster that encodes the *esxI*, *esxJ*, *ppe15* and *pe8* genes, it was found to be implicated in host inflammasome activation and in the secretion of ESX-5 PE_PGRS substrates [69]. As was discussed in the previous section, the PE/PPE proteins have been associated with *M. tb* virulence which makes ESX-5 an interesting target of study. 

### 3.2. ESX-3

The identification of *esx-3* as a paralogous *esx* locus suggests that it encodes a T7SS that may also be involved in *M. tb* pathogenesis. ESX-3 is a T7SS that transports its heterodimeric protein substrates PE5/PPE4 and EsxG/EsxH across the inner membrane [70].

In relation to its role in cellular physiology, ESX-3 is required for *M. tb* growth since its PE5/PPE4 substrate is mainly involved in metal homeostasis through mycobactin-mediated iron acquisition [71]. In contrast, its EsxG/EsxH substrate directly interacts with host endosomal sorting complexes required for transport proteins, which prevents phagosomal maturation and antigen presentation during macrophage infection with *M. tb* [72]. Recently, the structure of ESX-3 was solved by two independent groups [73,74], making it the second ESX secretion apparatus to be structurally characterized to date (discussed below). These structural findings enable further studies into the mechanism of substrate secretion by mycobacterial T7SS.

### 3.3. ESX-1

The ESX-1 secretion system is the most studied T7SS that was initially identified by comparative genomic analysis of *M. tb* and *M. bovis* BCG, an attenuated strain of *M. bovis* and the currently used TB vaccine. An early study using subtractive hybridization revealed that several distinct genomic regions were notably absent in BCG but were present in the virulent species, which have been termed region of differences (RD) [75]. Subsequent higher resolution studies confirmed these genomic differences between *M. tb* and BCG, while further identifying several other large sequence polymorphisms between BCG substrains during its course of in vitro evolution [35,76,77,78].

Of these genomic differences, region of difference 1 (RD1) is absent in all BCG substrains but is present in *M. tb* and *M. bovis*, suggesting that RD1 plays an important role in *M. tb* virulence and that the loss of this region contributed to the attenuation of BCG [76]. RD1 consists of a 9.5-kb DNA segment that encodes nine genes (*Rv3871* to *Rv3879c*) and is situated within the *esx-1* locus. *Rv3875* and *Rv3874* encode two secreted proteins, EsxA and EsxB, while adjacent genes in the RD1 locus encode components of the secretion apparatus. Inactivation of *esxA* and *esxB*, or of genes encoding components of the ESX-1 secretion apparatus, resulted in impaired growth of *M. tb* in macrophages and an attenuated phenotype in mouse models of infection, to an extent similar to that seen after deletion of the entire RD1 locus [79,80,81]. Another study on a clinical isolate of *M. tb* that harbored a frameshift mutation that inhibited EsxA secretion also led to an attenuated phenotype and resulted in decreased pro-inflammatory host immune responses [82]. In *M. marinum*, transposon inactivation of various genes at or near the RD1 locus resulted in the loss of EsxA/EsxB secretion, impaired growth of bacilli in macrophages, impaired ability to prevent phagolysosomal fusion, and reduced bacterial virulence in a zebrafish model of infection [83]. Collectively, these studies provide convincing evidence that the ESX-1 secretion system plays a major role in virulence.

On the other hand, reintroduction of ESX-1 into BCG does not restore full virulence and the RD1 deletion mutant of *M. tb* is still more virulent than BCG in long-term murine infection experiments, suggesting that additional genetic lesions may have contributed to the attenuation of BCG [84,85]. While EsxA and EsxB are secreted as a heterodimer in a 1:1 ratio [86], EsxA is considered to be a major effector of ESX-1 largely due to its membranolytic activity. Early studies found that purified EsxA, but not EsxB, caused lysis of artificial lipid bilayers [81]. However, the membranolytic activity of EsxA only occurred under acidic pH [87]. At neutral pH, ESX-1-mediated membrane lysis is contact-dependent and requires direct bacterial cell contact with host membranes [88]. This raises an intriguing possibility that EsxA may be delivered directly to the host membrane without being released into the extracellular milieu in order to exert its function. Another substrate of ESX-1 is the EspA/EspC heterodimer, which is encoded at a distal locus. The secretion of EspA/EspC is co-dependent on EsxA/EsxB [89,90]. Of particular interest is that EspC was found to form surface exposed filaments, which may provide a mechanism for contact dependent delivery of EsxA to the host membrane [91]. EspB is also a substrate of ESX-1 that has demonstrated membranolytic activity by assembling into heptameric ring-like structures that promote macrophage killing [92]. Despite being the most well-studied mycobacterial T7SS, the structure of the ESX-1 core membrane complex remains elusive.

### 3.4. Structure of ESX T7SS

Of the five ESX secretion apparatuses, the structure of ESX-5 of *M. xenopi* was the first to be solved by electron microscopy [62]. The core of this complex consists of four conserved ESX components—EccB5, EccC5, EccD5 and EccE5 in equimolar stoichiometry—that assembles into a hexameric pore in the inner membrane. The cytosolic C-terminus of EccC5 contains three ATPase domains and a flexible domain of unknown function (DUF) that is adjacent to its N-terminal transmembrane domain. Due to the flexible nature of the DUF, high-resolution analysis of the interaction between ESX-5 and its substrates could not be characterized. EccD5 is embedded in the inner membrane and makes small contact with the periplasm. On the periplasmic face of the inner membrane are the N-terminus of EccB5 and the C-terminus of EccE5 that are anchored to the membrane through transmembrane domains. It has been shown that a subset of PE/PPE substrates are transported by ESX-5 to modulate the composition of the cell wall [65], which may involve substrate contact with EccC5 and subsequent ATP hydrolysis to induce conformational changes in the ESX-5 apparatus to drive PE/PPE protein secretion.

The second ESX complex that was recently structurally characterized by cryogenic electron microscopy is ESX-3 of the model organism *M. smegmatis* [73,74]. The composition of its core includes four conserved components of the ESX machinery—EccB3, EccC3, EccD3 and EccE3, in a 1:1:2:1 stoichiometry—that form a pair of stable protomers which assemble into a proposed hexameric structure in the inner membrane. On the cytosolic face of the inner membrane, EccC3 comprises a flexible arrangement of four ATPase domains that are linked to the membrane through a region termed the stalk domain. Of these four ATPase domains, one has been attributed to a DUF that exists adjacent to the stalk domain that is necessary for secretion. The EccD3 dimer is embedded in the inner membrane and makes contact with a transmembrane helix of EccE3 to form a stable, rigid core. EccB3 is mainly located on the periplasmic face of the inner membrane, but it also traverses through the membrane and makes contact with the stalk domain of EccC3. It was postulated that once substrate binds to EccC3 of the ESX-3 complex, ATP hydrolysis occurs in the DUF that results in conformational changes in both the stalk domain and EccB3 to transport proteins from the cytosol to the periplasmic space.

**Figure 2:**
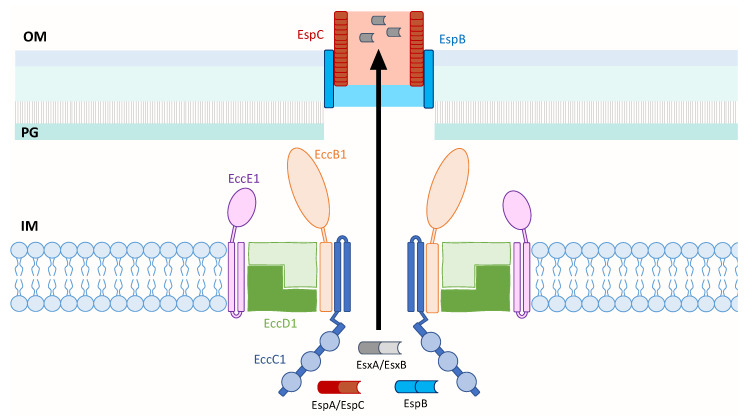
Model of the *M. tb* ESX-1 core membrane complex that is likely composed of EccB1, EccC1, EccD1 and EccE1 in a 1:1:2:1 ratio. EccC1 is shown with a flexible domain adjacent to the IM, followed by three ATPase domains represented by blue circles. When ESX-1 substrates contact EccC1, ATP hydrolysis occurs and is likely followed by conformational changes in the secretion apparatus to enable protein transport across the IM. The secreted EspB and EspC proteins may form an extended channel through the OM and into the extracellular milieu to transport EsxA across the OM. Inner membrane (IM); peptidoglycan (PG); outer membrane (OM).

Although the structure of the ESX-1 secretion system remains poorly characterized despite being one of the most studied T7SS, what is known about its secretion machinery is that it consists of an EccC1 ATPase [93], a membrane-bound mycosin serine protease that has dual function in processing secreted protein substrates [94] and stabilizing the membrane complex [95], as well as several other transmembrane proteins [60]. In light of its major role in mycobacterial virulence despite its unknown structure, the homology between ESX-1, ESX-3 and ESX-5, and the solved structures of ESX-3 and ESX-5 can provide valuable insight into a structural model of ESX-1 (Figure 2).

Similar to the membrane localization of the solved ESX complexes, this model of ESX-1 of *M. tb* depicts its core secretion apparatus in the inner membrane. The core likely consists of the four ESX conserved components—EccB1, EccC1, EccD1 and EccE1—in a 1:1:2:1 stoichiometry. This is based on the model of ESX-3 of *M. smegmatis* that shares 40.4% to 74.7% sequence identity of its EccB3 to EccE3 components with those of *M. tb* [73], and a structural study that showed dimerization of the cytoplasmic domain of EccD1 of ESX-1 [96]. These components are depicted to assemble into a pair of protomers that could assemble into a hexameric or larger multimeric pore similar to ESX-5 [62]. EccC1 likely forms the pore and contains a flexible region adjacent to the inner membrane that is followed by three known ATPase domains [92]. EccB1 is shown to adopt a fork-like structure on the periplasmic face, and both the periplasm-exposed EccB1 and EccE1 components are anchored to the inner membrane by transmembrane domains. Upon substrate binding to EccC1, it is postulated that ATP hydrolysis occurs that is followed by conformational changes in the ESX-1 secretion apparatus to enable protein transport across the inner membrane. The secreted substrates, EspB and EspC, may be responsible for transporting the effectors across the outer membrane to the external media or even directly to the host membrane upon cell-to-cell contact. This model of ESX-1 draws in components of the solved structures of ESX-3 and ESX-5 to gain insight into the structure of ESX-1 and the transport mechanism of its substrates, which still remains elusive.

## 4. Conclusions

*M. tb* and related mycobacterial pathogens use a complex assortment of virulence factors to cause disease in their hosts, which are not limited to surface-exposed lipids and secreted proteins. It was highlighted in this review that the identification and characterization of many surface-exposed lipids in the mycobacterial cell wall, including PDIMs, PGLs, and SLs, have provided valuable insight into the molecular basis of the interactions between mycobacteria and the host cell at the host–bacterium interface. The recently solved structures of ESX-3 and ESX-5 of the ESX secretion systems have also provided a framework for future studies involving ESX-1 that aim to elucidate the complete structure of its secretion apparatus, which remains unknown since its identification in 1996 [75].

Although much knowledge has been gained from the studies mentioned in this review, the methods used to identify mycobacterial virulence factors often begin with a global approach, such as transposon mutagenesis, to determine candidate virulence factors. Upon the identification of candidate virulence factors, it is important to perform validation studies using single-gene knockout mutants to understand the effects of the candidate genes or cellular components in an in vivo infection model. Future studies that aim to characterize novel virulence factors must also confirm the presence of PDIMs in their recombinant clones and associated parent strains, since the commonly used laboratory wild-type strain of *M. tb* H37Rv is highly susceptible to losing the ability to synthesize PDIMs over many passages in vitro [97]. Moreover, an increasing amount of evidence suggests that the virulent properties of pathogenic mycobacteria do not stem from the effects of a single gene or cellular component, but are rather a cumulative effect of many virulence factors.
